# Multi-curie production of gallium-68 on a biomedical cyclotron and automated radiolabelling of PSMA-11 and DOTATATE

**DOI:** 10.1186/s41181-020-00114-9

**Published:** 2021-01-07

**Authors:** Helge Thisgaard, Joel Kumlin, Niels Langkjær, Jansen Chua, Brian Hook, Mikael Jensen, Amir Kassaian, Stefan Zeisler, Sogol Borjian, Michael Cross, Paul Schaffer, Johan Hygum Dam

**Affiliations:** 1 Department of Nuclear Medicine, Odense University Hospital, Kløvervænget 47, 5000 Odense, Denmark; 2 Department of Clinical Research, University of Southern Denmark, Odense, Denmark; 3 ARTMS, Vancouver, BC Canada; 4 The Hevesy Laboratory, Health Technology, Technical University of Denmark, Roskilde, Denmark; 5 Life Sciences, TRIUMF, Vancouver, BC Canada

**Keywords:** Gallium-68, Cyclotron, Accelerator, DOTATATE, PSMA-11, Solid target, Targetry

## Abstract

**Background:**

With increasing clinical demand for gallium-68, commercial germanium-68/gallium-68 ([^68^Ge]Ge/[^68^Ga]Ga) generators are incapable of supplying sufficient amounts of the short-lived daughter isotope. In this study, we demonstrate a high-yield, automated method for producing multi-Curie levels of [^68^Ga]GaCl_3_ from solid zinc-68 targets and subsequent labelling to produce clinical-grade [^68^Ga]Ga-PSMA-11 and [^68^Ga]Ga-DOTATATE.

**Results:**

Enriched zinc-68 targets were irradiated at up to 80 µA with 13 MeV protons for 120 min; repeatedly producing up to 194 GBq (5.24 Ci) of purified gallium-68 in the form of [^68^Ga]GaCl_3_ at the end of purification (EOP) from an expected > 370 GBq (> 10 Ci) at end of bombardment. A fully automated dissolution/separation process was completed in 35 min. Isolated product was analysed according to the Ph. Eur. monograph for accelerator produced [^68^Ga]GaCl_3_ and found to comply with all specifications. In every instance, the radiochemical purity exceeded 99.9% and importantly, the radionuclidic purity was sufficient to allow for a shelf-life of up to 7 h based on this metric alone. Fully automated production of up to 72.2 GBq [^68^Ga]Ga-PSMA-11 was performed, providing a product with high radiochemical purity (> 98.2%) and very high apparent molar activities of up to 722 MBq/nmol. Further, manual radiolabelling of up to 3.2 GBq DOTATATE was performed in high yields (> 95%) and with apparent molar activities (9–25 MBq/nmol) sufficient for clinical use.

**Conclusions:**

We have developed a high-yielding, automated method for the production of very high amounts of [^68^Ga]GaCl_3_, sufficient to supply proximal radiopharmacies. The reported method led to record-high purified gallium-68 activities (194 GBq at end of purification) and subsequent labelling of PSMA-11 and DOTATATE. The process was highly automated from irradiation through to formulation of the product, and as such comprised a high level of radiation protection. The quality control results obtained for both [^68^Ga]GaCl_3_ for radiolabelling and [^68^Ga]Ga-PSMA-11 are promising for clinical use.

## Background

In the 1960s, the first [^68^Ge]Ge/[^68^Ga]Ga-generators emerged and formed the early basis for modern, solid phase based generators of medical-grade gallium-68 (t_½_: 68 min) (Gleason 1960; Greene and Tucker 1961; Yano and Anger 1964). Since then, generators remained as a reliable method for supplying this PET radionuclide for the development of novel radiotracers and imaging techniques. With diagnostic and therapeutic breakthroughs, particularly those in prostate cancer and neuroendocrine tumour imaging, the demand for gallium-68 is rapidly outpacing the supply available from [^68^Ge]Ge/[^68^Ga]Ga-generators.

Direct cyclotron production of gallium-68 using the ^68^Zn(p,n)^68^Ga transformation on small medical cyclotrons provides access to clinically relevant quantities of the gallium-68 isotope. Liquid targets, using acidic solutions containing zinc-68 salts, are suitable for a small number of patient doses, but this method also carries the inherent risk of cyclotron damage upon target failure. Solid targets are more reliable and provide much higher yields, but until recently commercial systems capable of transporting solid targets by remote operation were limited and were not compatible with many self-shielded medical cyclotrons currently installed.

We report here on a high-yield automated method of producing [^68^Ga]GaCl_3_ from solid targets and subsequent labelling to form [^68^Ga]Ga-PSMA-HBED-CC ([^68^Ga]Ga-PSMA-11) and [^68^Ga]Ga-DOTATATE. Quality control tests were performed in accordance to the draft Ph. Eur. monographs (Gallium (68Ga) PSMA-11 Injection 2018; Gallium (68Ga) Chloride (Accelerator-Produced) Solution for Radiolabelling 2017), which, after the work was completed, have been released in their final versions to which both the [^68^Ga]GaCl_3_ and [^68^Ga]Ga-PSMA-11 still comply.

## Materials and methods

### Materials

All water was obtained from a Milli-Q® Direct-Q 3 UV system (Merck Millipore). Ultrapur hydrochloric acid 30% (Merck) was used directly and diluted with water during target dissolution and gallium-68 purification. Hydroxamate-based resin (ZR Resin), di(2-ehtylhexyl)orthophosphoric acid-based resin (LN Resin) and trioctylphosphine-based resin (TK200 Resin) were used for [^68^Ga]GaCl_3_ purification (Triskem International, Rennes, France). Labelling was performed with PSMA-11 and DOTATATE (ABX, Dresden, Germany).

### Irradiation

Gallium-68 was produced via the ^68^Zn(p,n)^68^Ga nuclear reaction using highly enriched metallic zinc-68 solid targets (ARTMS, Vancouver, Canada, zinc-68: > 98.2%, Ø10 mm, 230–315 mg on water cooled silver backing). The zinc-68 solid targets were transferred and irradiated using the ARTMS QUANTM Irradiation System® (“QIS”, ARTMS, Vancouver, Canada) on a GE PETtrace 880 Cyclotron (GEMS PET Systems AB, Uppsala, Sweden) using a helium-cooled aluminium foil energy degrader to achieve a proton incident energy of 13.0 MeV on target. The targets were irradiated using proton beam currents of up to 80 µA for up to 2 h. Prior to applying the maximum beam current of 80 µA on target, a series of beam current ramping experiments were performed on natural zinc targets. The irradiations were performed with increasing beam currents in steps of 5–10 µA (with all other parameters kept unchanged) followed by visual inspection of the irradiated target in each step.

### Target dissolution

**Fig. 1:**
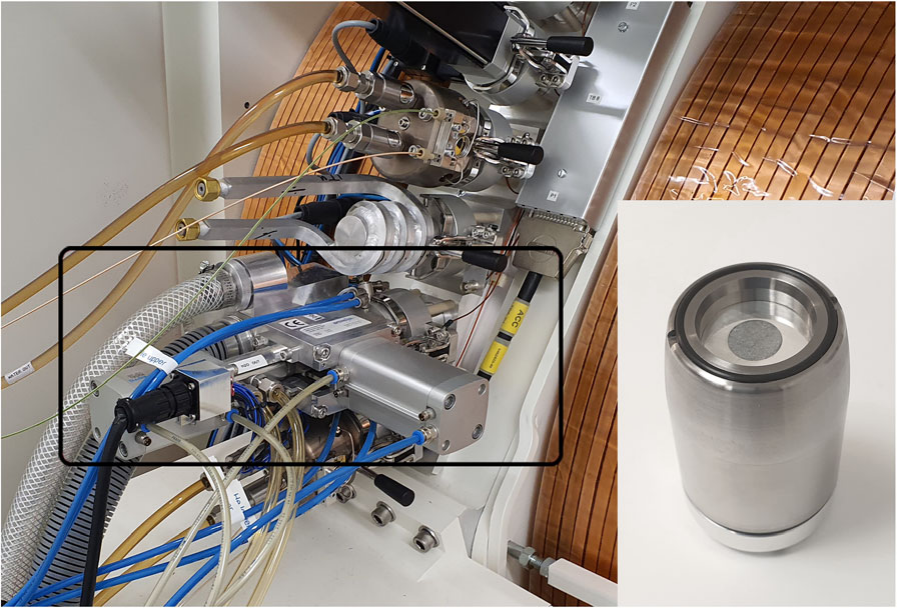
The ARTMS QUANTM Irradiation System® on the GE PETtrace (rectangular box) allowing for automated pneumatic transfer of the water-cooled target capsule (**insert**) containing the enriched zinc-68 on silver backing

After irradiation, the target capsule (Fig. 1) was pneumatically transferred to a hot cell by the QIS. The zinc-68 targets were then dissolved using 2 mL of hot (approx. 90 °C) 30% HCl on a QIS Dissolution System. Evolved hydrogen was vented.

### Purification

**Fig. 2:**
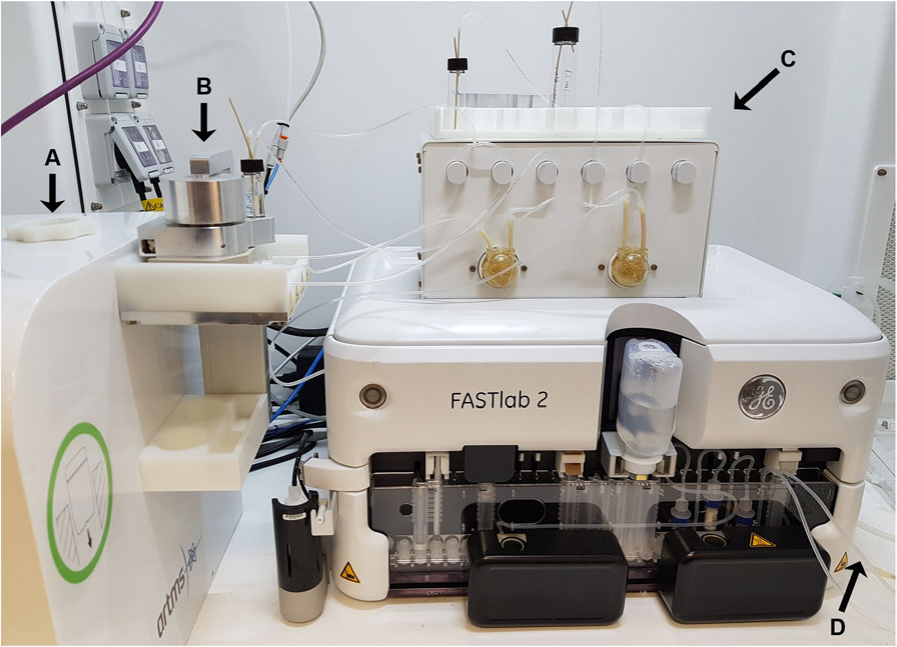
ARTMS receive station (**a**), zinc-68 dissolution cell (**b**) and dissolution box (**c**) coupled to the GE FASTlab 2 Developer synthesis module with gallium-68/zinc-68 separation cassette installed. The [^68^Ga]GaCl_3_ outlet (**d**) was connected to the Modular-Lab PharmTracer (not shown) for automated radiolabelling of PSMA-11

The dissolved target solution containing [^68^Ga]GaCl_3_ and [^68^Zn]ZnCl_2_ was automatically transferred to a GE FASTlab 2 Developer synthesis module for separating the gallium-68 from the target material by three solid phase extraction columns (Fig. 2). First, the target solution was pumped through a column containing 250 mg ZR resin. 15 mL of 30% HCl were used to elute remaining zinc-68 from the ZR column, while gallium-68 was eluted from the column with 8 mL of 1 N HCl. The solution containing gallium-68 was pushed through the second column containing LN resin in order to bind Fe contaminants, while the eluted [^68^Ga]GaCl_3_ was directly loaded onto a third column containing TK200 resin. The TK200 resin was dried by purging with nitrogen, then eluted to a product vial using 2.5 mL of 0.1 N HCl. The overall process of target dissolution and purification took approximately 35 min.

### Quality control gallium-68

Quality control of the produced [^68^Ga]GaCl_3_ was performed according to the draft Ph. Eur. monograph on accelerator produced [^68^Ga]GaCl_3_ (Gallium (68Ga) Chloride (Accelerator-Produced) Solution for Radiolabelling 2017).

Gamma-ray spectrometry and radionuclidic purity assays were performed using a calibrated Canberra Broad Energy Ge detector (BE2020, Canberra) equipped with Genie 2000 software. Half-life was determined by repeated measurements in a Capintec dose calibrator. pH was determined by pH sticks (Merck MColorpHast pH 0–6.0). The silver nitrate precipitation assay was performed by adding 5 µL gallium-68 chloride solution to 0.1 mL silver nitrate (17 g/L) to yield a white precipitate. The solid phase extraction (SPE) assay was done by application of the Phenomenex Strata-X-C cation exchange cartridge (33 µm, 10 mg/mL) preconditioned with 0.5 mL ethanol, then 1 mL 1.03 g/l HCl. The gallium-68 solution was slowly loaded onto the cartridge, the liquids collected and the column dried with air. The SPE column was washed with 1 mL HCl (1.03 g/l) and the liquids were collected. The column was then eluted with 1 mL 2% (v/v) HCl (1.03 g/l) in acetone and dried with air to collect the final eluate. The quantity of radioactivity in each of the different fractions and the column were measured in the dose calibrator. Radiochemical purity was assessed by thin-layer chromatography on an Agilent ITLC-SG plate: A sample of the gallium-68 DTPA solution was added to the ITLC plate, which was eluted in 1:1 ammonium acetate (77 g/l): methanol over at least 7 cm. The radioactivity distribution was evaluated on a MiniGita TLC scanner (Elysia-Raytest). Contents of iron and zinc were determined with semi-quantitative test strips (Quantofix™, Machery-Nagel) by comparing the colour change of the reactive pad against multi-stage colour scales. Further, the metal contents of decayed samples of the gallium-68 solution were quantified as described in draft Ph. Eur. monograph (Gallium (68Ga) Chloride (Accelerator-Produced) Solution for Radiolabelling 2017) by ICP-OES in an argon plasma (Thermo iCAP 6000 ICP Spectrometer, Thermo Scientific, Waltham, MA, USA). The contents of bacterial endotoxins was determined on an Endosafe Nexgen-PTS™ by adjusting and diluting the pH of a sample to pH 6–8 with NaOH (10 mM, in endotoxin free water (Charles River)) for minimum inhibition of the endotoxin assay before adding the solutions to the single use cartridge.

### Radiolabelling of DOTATATE with gallium-68

Zinc-68 targets containing 230–315 mg zinc-68 were irradiated with 50–70 µA for 0.5–1 h and the produced gallium-68 was separated from target material as described above. Radiolabelling of DOTATATE with gallium-68 was performed manually. For labelling with low activity levels (< 1 GBq, n = 3), 100 µl of the purified gallium-68 solution was used, while approximately 900 µl was used for the single 3.2 GBq labelling. For the low activity labelling, the [^68^Ga]GaCl_3_ was microwave heated (CEM PETwave) to 90 °C for 2 min. with 50 µg DOTATATE (34.8 nmol) in 125 µl acetate buffer (pH 4.6, Fluka) and 20 µl ethanol added. For higher activity labelling (3.2 GBq), the [^68^Ga]GaCl_3_ solution was mixed with 500 µl acetate buffer and then preheated to 95 °C before adding 500 µg of DOTATATE (348 nmol) in 100 µl acetate buffer. The solution was kept at 95 °C for 10 min. In all cases the labelling yield and radiochemical purity was assessed by ITLC (Biodex 150–771, 0.1 M sodium citrate, pH 5) and radio-HPLC on a Phenomenex Jupiter 300A (150 × 4.6, 5 µm) C18 column in water + 0.1% TFA (**A**) and acetonitrile (**B**); 0–2 min. 14% **B**, 2–10 min. 14 to 63% **B** at 1 mL/min. and UV detection at 220 nm.

### Radiolabelling of PSMA-11 with gallium-68

**Fig. 3:**
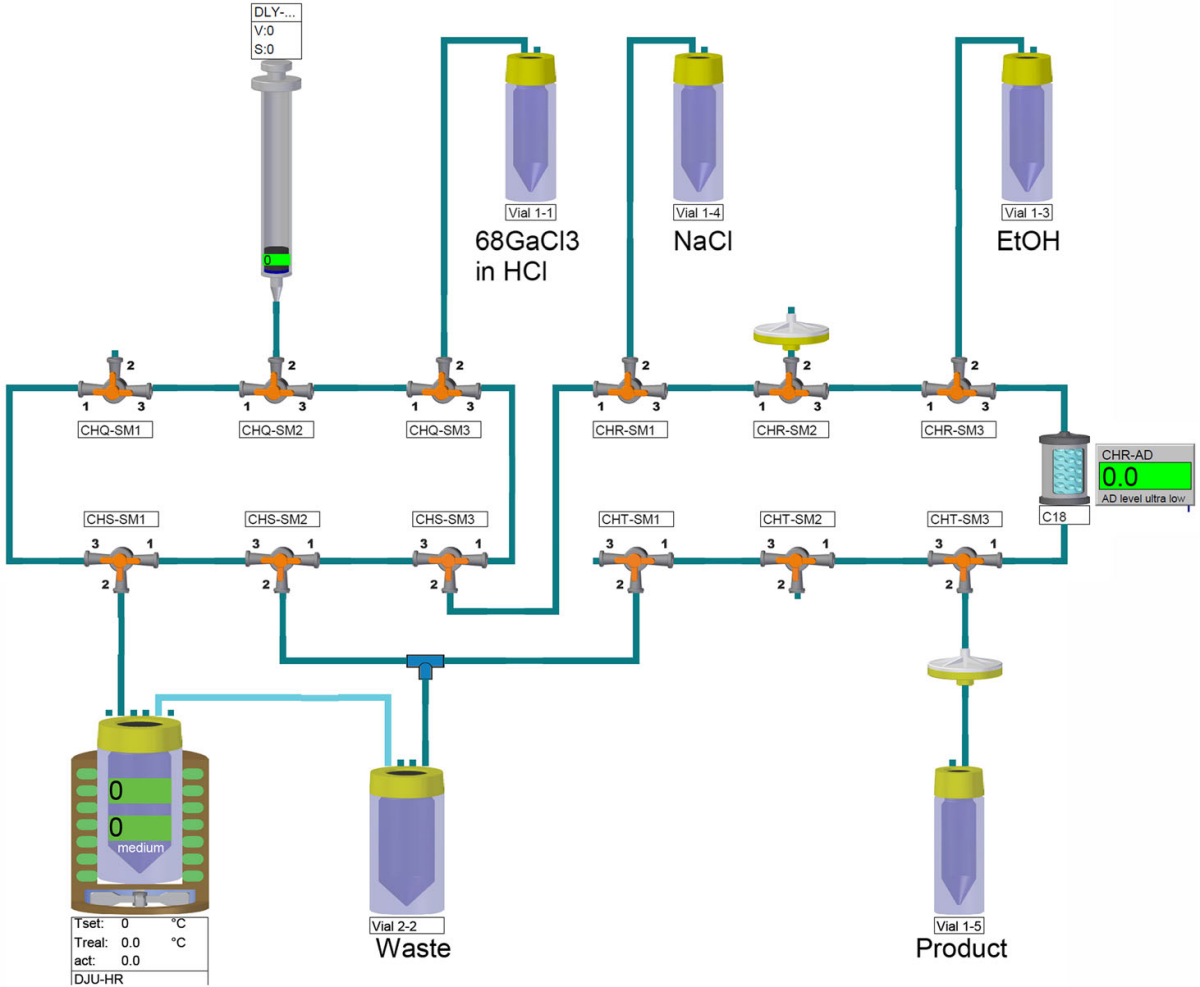
Overview of the automated synthesis cassette for radiolabelling of PSMA-11 using the Modular-Lab PharmTracer by Eckert & Ziegler

Zinc-68 targets containing 230–315 mg zinc-68 were irradiated with 80 µA for 2 h and the produced gallium-68 was separated from the target material as described above. Radiolabelling of PSMA-11 with [^68^Ga]GaCl_3_ was achieved with a single use kit on an automated Modular-Lab PharmTracer (Eckert & Ziegler) in direct connection to the FASTlab synthesizer. The standard cassette was modified to accommodate accelerator-produced gallium-68 radiolabelling Fig. 3, *i.e.* the cation exchange column was removed and the module was programmed to withdraw the gallium-68 solution from the FASTlab product vial and deliver 1.0 mL (out of the 2.2–2.4 mL total produced gallium-68 chloride solution) to the reactor. The reactor was also loaded with 50 mg of sodium ascorbate, 100 nmol PSMA-11 (ABX, Germany) and 1.0 mL sodium acetate buffer, pH 4.6. During the labelling, the reactor was heated to 95 °C for 600 s after which the solution was diluted with 3 mL deionized (DI) water and loaded onto a preconditioned SepPak C18 light column. The reactor was further washed with 3 mL DI water and loaded onto the C18 light column, which was washed with 3 mL of DI water. The column was eluted with 2 mL 50% (v/v) ethanol followed by 4 mL DI water into a product vial containing 50 mg sodium ascorbate in 6 mL phosphate buffered saline to yield a final product volume of approximately 12 mL containing the [^68^Ga]Ga-PSMA-11 at the end of synthesis (EOS).

For quality control, an aliquot was analysed by HPLC on a Phenomenex Kinetex XB-C18 column (150 × 4.6, 5 µm, 1 mL/min) applying a gradient program (A:B, DI water + 0.1% TFA: acetonitrile) similar to that specified in the Ph. Eur [^68^Ga]Ga-PSMA-11 monograph draft, *i.e*. 0–0.5 min. 5% B then 5–40% B from 0.5 to 10 min (Gallium (68Ga) PSMA-11 Injection 2018). Also, the amounts of unlabelled and colloid gallium-68 were determined by TLC on silica gel on glass backing plates eluted in either 0.1 M sodium citrate, pH 5 or ammonium acetate in DI water (77 g/l):methanol 1:1, respectively. pH was checked by a calibrated pH meter.

## Results

### Targetry and [^68^Ga]GaCl_3_ productions

During beam current ramping experiments, we determined that the zinc targets were capable of withstanding beam currents of at least 80 µA of 13 MeV protons for 2 h, which is equivalent to approximately 1 kW of power deposited in the zinc target material.

Irradiations performed under these conditions (80 µA for 120 min) produced up to 194 GBq (5.24 Ci) of purified [^68^Ga]GaCl_3_ at the end of purification (EOP) from an expected > 370 GBq (> 10 Ci) gallium-68 at end of bombardment (EOB). The fully automated dissolution/radiochemical separation was performed in 35 min. Multiple productions were analysed according to the draft Ph. Eur. monograph on accelerator produced gallium-68 and found to comply with all requirements (Table 1). In all productions the radiochemical purity of the gallium-68 chloride was above 99.9% and the radionuclidic purity (RNP) above 99.85% at EOP. That is, the combined content of co-produced gallium-66 and gallium-67 was in the range 0.029–0.15% of the total activity at EOP, *i.e*., much lower than the 2% limit given in the draft Ph. Eur. monograph. Based on RNP alone, this allows for a shelf-life (according to EU requirements) of up to 7 h of the purified [^68^Ga]GaCl_3_ or of a gallium-68-labelled radiopharmaceutical prepared using the solution. Of particular importance is the low iron and zinc contents achieved in the purified [^68^Ga]GaCl_3_ product (Table 1), being orders of magnitude lower than the limits given in the draft Ph. Eur. monograph. That is, only 3.6 ng/GBq iron and 58 ng/GBq zinc were present in the [^68^Ga]GaCl_3_ batch with the highest activity.

**Table 1:** Overview of selected [^68^Ga]GaCl_3_ productions with associated quality control results analysed according to draft Ph. Eur. monograph on accelerator produced gallium-68. Limits given by the draft Ph. Eur. monograph are shown in parentheses. ND: Not determined. WP: White precipitate. EOP: End of purification

Production run	1	2	3	4
Zinc-68 mass	315 mg	230 mg	240 mg	300 mg
Beam current & time	50 µA, 30 min	70 µA, 30 min	80 µA, 2 h	80 µA, 2 h
Charge (µAh)	25	36.2	163.4	164
GBq@EOP	37	51	152	194
Appearance (Clear & colorless)	C&C	C&C	C&C	C&C
γ-Spectrometry (Confirmed ID by 511 & 1077 keV)	Confirmed	Confirmed	ND	Confirmed
T½ (62―74 min.)	66.2	71.2	66.4	67.1
Solid Phase Extraction (> 90% of total radioactivity)	91.3%	95.0%	ND	93.8%
Precipitation with AgNO_3_	WP	WP	WP	WP
pH (< 2)	< 2	< 2	< 2	< 2
Contents of Fe (< 10 µg/GBq)	0.037 µg/GBq	0.0077 µg/GBq	0.0074 µg/GBq	0.0036 µg/GBq
Contents of Zn (< 10 µg/GBq)	0.36 µg/GBq	0.85 µg/GBq	0.18 µg/GBq	0.058 µg/GBq
Bacterial endotoxins (< 175 IU/ V)	< 1.75 IU/mL	< 2 IU/mL	ND	< 1.85 IU/mL
γ-Spectrometry (> 98% Ga-68)	99.95%	99.89%	99.85%	99.97%
Contents of Ga-66/67 (< 2%)	0.047%	0.11%	0.15%	0.029%
Other radionuclidic impurities (< 0.1%)	Not detected	Not detected	Not detected	Not detected
TLC (> 95% Ga^3+^)	100%	99.9%	100% (@ + 24 h)	99.9%

### Radiolabelling of DOTATATE

To test the purity of the produced gallium-68 chloride, initial manual radiolabelling tests of DOTATATE were performed (Table 2). The [^68^Ga]Ga-DOTATATE was produced in high yields (> 95%) and in clinically acceptable apparent molar activities (AMA) of 9–25 MBq/nmol (non-optimized). Up to 3.2 GBq of [^68^Ga]Ga-DOTATATE was produced. No attempts were made to increase the activity due to radiation protection issues of the manual method used.

**Table 2:** Produced activities at end of synthesis (EOS, not decay corrected) and associated AMA and purities for [^68^Ga]Ga-PSMA-11 and [^68^Ga]Ga-DOTATATE. n: Amount of peptide used for radiolabelling. TLC and TLC (colloid) denominates the RCP of [^68^Ga]Ga-PSMA-11 from analysis for free and colloid gallium-68, respectively

**[**^**68**^**Ga]Ga-PSMA-11**					
**Activity at EOS**	**n**	**AMA at EOS**	**RCP (HPLC)**	**TLC**	**TLC (colloid)**
**GBq**	**nmol**	**MBq/nmol**	**%**	**%**	**%**
39.8	100	398	99.9	100	99.7
42.9	100	429	99.8	100	99.9
72.2	100	722	98.2	100	99.8
		***Average:***	***99.3***	***100***	***99.8***
		*Std.dev.:*	*1.0*	*0*	*0.1*
**[**^**68**^**Ga]Ga-DOTATATE**					
**Activity at EOS**	**n**	**AMA at EOS**	**RCP (HPLC)**	**ITLC**	
**GBq**	**nmol**	**MBq/nmol**	**%**	**%**	
0.87	34.8 (50 µg)	25	97.3	98.4	
0.88	34.8 (50 µg)	25	98.2	98.4	
0.75	34.8 (50 µg)	23	99.7	99.6	
3.22	348 (500 µg)	9.2	95.4	97.1	
		***Average:***	***97.7***	***98.4***	
		*Std.dev.:*	*2.2*	*1.2*	

### Radiolabelling of PSMA-11

Radiolabelling of PSMA-11 was performed fully automated on a Modular-Lab PharmTracer module, hence much higher gallium-68 activities could be used. The resulting [^68^Ga]Ga-PSMA-11 activities and corresponding RCP and AMA are summarized in Table 2. Up to 72.2 GBq (approximately 2 Ci) of [^68^Ga]Ga-PSMA 11 was produced fulfilling key requirements in the draft Ph. Eur. monograph on [^68^Ga]Ga-PSMA-11 injection such as RCP, RNP and colloid formation as judged by radioHPLC, ITLC and gamma spectrometry (Gallium (68Ga) PSMA-11 Injection 2018). The amounts of unlabelled and colloid gallium-68 were determined to 0% and 0.12–0.35%, respectively. By HPLC, the RCPs were determined to be 99.3 ± 1.0%. The pH was measured to be between 7.0 and 7.6. The [^68^Ga]Ga-PSMA-11 was produced with an AMA of up to 722 MBq/nmol at EOS for the highest obtained activity of 72.2 GBq. The amount of unlabelled [^68^Ga]GaCl_3_ separated by the SepPak C18 purification after the labelling step constituted only 2.3 ± 0.2% of the total activity at EOS and was as such disregarded. Hence, the AMA of the [^68^Ga]GaCl_3_ can be estimated to be approximately the same or higher than the AMA for the [^68^Ga]Ga-PSMA-11. The stability of the [^68^Ga]Ga-PSMA-11 was evaluated over 2 h post EOS for this batch and no change was seen in RCP during this time (98.2% at EOS, 98.6% at EOS + 2 h) but a minor increase in colloid formation from 0.18% to 0.34% was observed. Hence, the [^68^Ga]Ga-PSMA-11 was stable for at least 2 h despite the very high initial activity.

## Discussion

At present, the supply of gallium-68 for medical imaging is primarily based on the [^68^Ge]Ge/[^68^Ga]Ga-generator. As these commercial generators can deliver only a limited amount of activity, and the demand for gallium-68 is increasing with the commercialization of kit-based radiopharmaceuticals, e.g. NETSPOT (DOTATATE), SomakitTOC (DOTATOC) and Illumet (PSMA-11), a future shortage of generators and gallium-68 may occur. Herein, we describe a high-yielding, automated production of this important isotope by irradiating solid targets of enriched ^68^Zn metal on a biomedical cyclotron. This approach may serve as an important supplement to meet future demand.

By application of silver targets with enriched zinc-68 from ARTMS on the ARTMS QIS target system (Fig. 1), gallium-68 was produced by 13 MeV proton irradiation at beam currents up to 80 µA. After irradiation, the solid target was pneumatically transferred from the cyclotron to a dissolution box in a hot cell (Fig. 2). The automated transfer comprises a very favourable and necessary feature as very high levels of radioactivity from estimated > 370 GBq gallium-68 on the target were produced at EOB. Nelson et al. recently reported on a new method for cyclotron-based gallium-68 production by irradiation of zinc-68 pellets with production yields up to 37.5 ± 1.9 GBq on target (non-purified) (Nelson et al. 2020). However, their method involves manual collection of the irradiated target and subsequent transport of the target to the hot cell in a lead shield by the operator. Such a method would lead to radiation protection issues if much higher radioactivities were routinely produced, as demonstrated in the current study. Here, automated target transfer and subsequent automatic separation and radiolabelling are mandatory to avoid excessive radiation exposure to the operator.

Samples of several productions were analysed according to the draft Ph. Eur. monograph on accelerator produced gallium-68 (Gallium (68Ga) Chloride (Accelerator-Produced) Solution for Radiolabelling 2017). The results of the tests are summarized in Table 1. Notably, the RCP and RNP were high and only minor amounts of gallium-66/67 were present, while no other radionuclidic impurities were detected by gamma spectrometry. Prolonged irradiation and higher beam currents expectedly improved the RNP and the AMA as judged by HPGe detection and ICP-OES, only 3.6 ng/GBq Fe and 58 ng/GBq Zn were present in the [^68^Ga]GaCl_3_ batch with the highest activity. This is more than 30 times lower than the iron contents of 0.13 ± 0.07 µg/GBq Fe found by Nelson et al*.* and 0.11 ± 0.07 µg/GBq Fe found by Lin et al., which was the dominating metal impurity in these studies (Nelson et al. 2020; Lin et al. 2018). The improvement in the iron content in our method, which is highly important for obtaining a high AMA in subsequent labelling of radioconjugates, is due to the introduction of the second column containing LN resin in the separation, where Fe contaminants are bound. Importantly, the RNP was ≥ 99.89% and allowed for a shelf-life of the [^68^Ga]Ga chloride of up to 7 h based on RNP alone.

To estimate the purity before ICP-OES results were obtained, the produced [^68^Ga]Ga chloride was applied to radiolabel DOTATATE and PSMA-11. High labelling yield and AMA comparable to or higher than what is observed for the generator-produced isotope indicated a low amount of metallic impurities in the accelerator produced gallium-68. An Eckert-Ziegler synthesis module with a modified cassette and sequence directly connected to the separation module was applied to radiolabel PSMA-11 with very high levels of radioactivity (> 39.8 GBq). Several high activity productions with only 100 nmol of PSMA-11 were performed yielding up to 72.2 GBq [^68^Ga]Ga-PSMA-11 (uncorrected for decay; 23 min synthesis time), which was stabilized with 50 mg sodium ascorbate and 1 mL ethanol in a 12 mL volume. The radioconjugate was tested for stability up to two hours after EOS (by HPLC, colour, TLCs for free and colloid gallium) and found stable in this formulation and very high AMA. RCP was higher than 98.5% after 2 h.

In terms of patient doses, a batch of 70 GBq [^68^Ga]Ga-PSMA-11 would represent at least 10 doses for 50 min/scan protocol for two simultaneously running PET-scanners. If PET scanner capacity is doubled to four scanners, the batch could deliver up to 20–25 patient doses of [^68^Ga]Ga-PSMA-11 from a single solid target based gallium-68 production.

Otherwise, a radioconjugate (or purified [^68^Ga]GaCl_3_) of this stability and AMA could be transported to decentralized radiopharmacies possibly superseding the need for expensive generators on a continuous basis. Also, a generator carries the risk of germanium-68 breakthrough to the final product and eventually becomes long-lived radioactive waste (half-life of germanium-68: 271 days) after about 400 elutions, which needs to be disposed of. In comparison, no long-lived radioactive waste is produced by the cyclotron production route if the silver backing (containing some cadmium-109) is reused. Conversely, if only a limited amount of gallium-68 activity is needed (*i.e.* a few patient doses), a generator is much less technically demanding, very reliable and does not require trained personnel to perform separation and radiolabelling on synthesis modules or major capital investment in a cyclotron facility. Liquid cyclotron targets where acidic solutions containing zinc-68 salts are bombarded with protons, provide an alternative solution to get a small number of patient doses but the reported production yields of < 10 GBq at EOB or approximately 5 GBq at EOP are inferior to the solid target route (Pandey et al. 2019; Alves et al. 2018; Pedersen et al. 2019; do Carmo et al. 2020; Melissa et al. 2020). This method also carries the inherent risk of acids damaging targets and potentially cyclotron systems and require continuous target maintenance.

Other studies on solid target based [^68^Ga]GaCl_3_ productions have been published recently (Schweinsberg et al. 2019). Alnahwi et al. reported on the production of 145 GBq gallium-68, decay-corrected to EOB, by proton irradiation of pressed zinc-68 targets with 35 µA on target (Alnahwi et al. 2020). However, the use of targets made from pressed zinc powder with expectedly reduced heat transfer compared to solid zinc limits the maximum power density that can be absorbed in the target and thus, the maximum beam current. The resulting purified activity available at EOP for radiolabelling was not stated, although the authors reported an AMA of the produced [^68^Ga]GaCl_3_ for a 20 min irradiation to be 28.3 ± 6.8 GBq/µmol. Radiolabelling of 21 nmol DOTATATE with approximately 555 MBq [^68^Ga]GaCl_3_ at time of radiolabelling resulted in a labelling efficiency of 95 ± 1.6%, which is similar to the results obtained in the current study. However, the AMA of the [^68^Ga]Ga-DOTATATE is not reported by the authors but can be estimated to be approximately 26 MBq/nmol at time of labelling. This is in line with the AMA we observed for [^68^Ga]Ga-DOTATATE using manual labelling procedures but inferior to the AMA of [^68^Ga]Ga-PSMA-11 (and consequently the AMA of the [^68^Ga]GaCl_3_) in our automated syntheses reaching as high as 722 MBq/nmol (range 398–722 MBq/nmol). In comparison, the AMA obtained by Nelson et al. for [^68^Ga]Ga chloride using DOTA-labelling was reported as 9.5 ± 1.3 GBq/µmol (Nelson et al. 2020), and thus inferior to the AMA obtained in the present study.

In summary, the production method proposed here, to our knowledge, signifies a record high production of purified [^68^Ga]GaCl_3_ and of [^68^Ga]Ga-PSMA-11 with high RNP, RCP and very high AMA. Furthermore, the highly automated method ensures a low radiation burden to the operator, despite the multi-Curie levels of radioactivity produced. As such, this production route features a favourable supplement to generator-produced gallium-68 capable of delivering tens of patient doses for gallium-68 tracers as demonstrated with record high quantities of [^68^Ga]Ga-PSMA-11.

## Conclusion

A high-yield method for direct cyclotron production of [^68^Ga]GaCl_3_ has been demonstrated, leading to record high purified gallium-68 activities (194 GBq at EOP) and subsequent labelling of PSMA-11 (72.2 GBq) and DOTATATE. For production of [^68^Ga]Ga-PSMA-11 the entire process was highly automated from irradiation to formulation of the product and as such comprised a high level of radiation protection. The quality control results obtained for both [^68^Ga]GaCl_3_ for radiolabelling and [^68^Ga]Ga-PSMA-11 and [^68^Ga]Ga-DOTATATE are promising for clinical use.

## Data Availability

The datasets used and/or analyzed during the current study are available from the corresponding author on reasonable request.

## Abbreviations

AMA: Apparent molar activities
DI: Deionized
EOB: End of bombardment
EOP: End of purification
EOS: End of synthesis
HPGe: High purity germanium detector
ICP-OES: Inductively coupled plasma optical emission spectrometry
ITLC: Instant thin layer chromatography
LN Resin: Di(2-ehtylhexyl)orthophosphoric acid-based resin
PSMA-11: PSMA-HBED-CC
QIS: QUANTM Irradiation System®
RadioHPLC: Radio High-performance liquid chromatography
RCP: Radiochemical purity
RNP: Radionuclidic purity
SPE: Solid phase extraction
TFA: Trifluoroacetic acid
TK200 Resin: Trioctylphosphine-based resin
ZR Resin: Hydroxamate-based resin
